# Transesophageal Echocardiography Assisting in the Identification of Intraabdominal Bleeding During Cardiac Surgery

**DOI:** 10.7759/cureus.48105

**Published:** 2023-11-01

**Authors:** Asad Mansoor, Arun K Singhal, Satoshi Hanada

**Affiliations:** 1 Department of Anesthesia, University of Iowa Hospitals and Clinics, Iowa City, USA; 2 Department of Cardiothoracic Surgery, University of Iowa Hospitals and Clinics, Iowa City, USA

**Keywords:** focused assessment with sonography in trauma, diagnostic laparoscopy, chest tube, cardiac anesthesia, abdominal bleeding, transesophageal echocardiography (tee)

## Abstract

We present a case in which intraoperative transesophageal echocardiography (TEE) helped detect intraabdominal bleeding, a rare complication in cardiac surgery. A patient undergoing ascending aortic aneurysm and aortic valve repair had increasing vasopressor and transfusion requirement during sternal closure with TEE imaging revealing a nonspecific, hypoechoic fluid-like collection anterior to the stomach. Discussion between the anesthesiology and surgical teams prompted further investigation including a diagnostic laparoscopy which confirmed the presence of intraabdominal bleeding. Hemostasis was later achieved after identifying the source of bleeding from a pre-peritoneal vein and associated peritoneal defect adjacent to a mediastinal chest tube placed earlier in the operation.

## Introduction

Transesophageal echocardiography (TEE) is an important monitoring tool used intraoperatively, when indicated. It plays a significant role in both surgical and anesthetic decision-making. Practice guidelines published by the American Society of Echocardiography emphasize the importance of communication between the surgical and anesthesiology teams to effectively guide intraoperative management [1]. Although most would agree that such communication is crucial in the operating room, execution of effective communication between the anesthesiologists and surgeon is not always so simple. Raemer et al. performed a simulation-based randomized controlled experiment, identifying reasons why an anesthesiologist may decide not to speak up [2]. At the conclusion of the study, "uncertainty about the issue" was identified as one of the most common hurdles preventing anesthesiologists from speaking up. The following case is a prime example where the anesthesiologist spoke up despite uncertainty surrounding the presence of a suspected intraabdominal hemorrhage, an extremely rare complication contributing to hemodynamic instability at the conclusion of ascending aortic aneurysm and aortic valve repair surgery.

This article was previously presented as a poster presentation at the 2022 American Society of Anesthesiologists Meeting.

## Case presentation

A 65-year-old woman with systolic heart failure (ejection fraction=46%), obesity (BMI=43 kg/m^2^), severe aortic regurgitation, and ascending aortic aneurysm presented for open ascending aortic aneurysm and aortic valve repair. She originally presented to the hospital one week prior to surgery with an acute heart failure exacerbation requiring hospitalization for medical optimization. 

**Figure 1:**
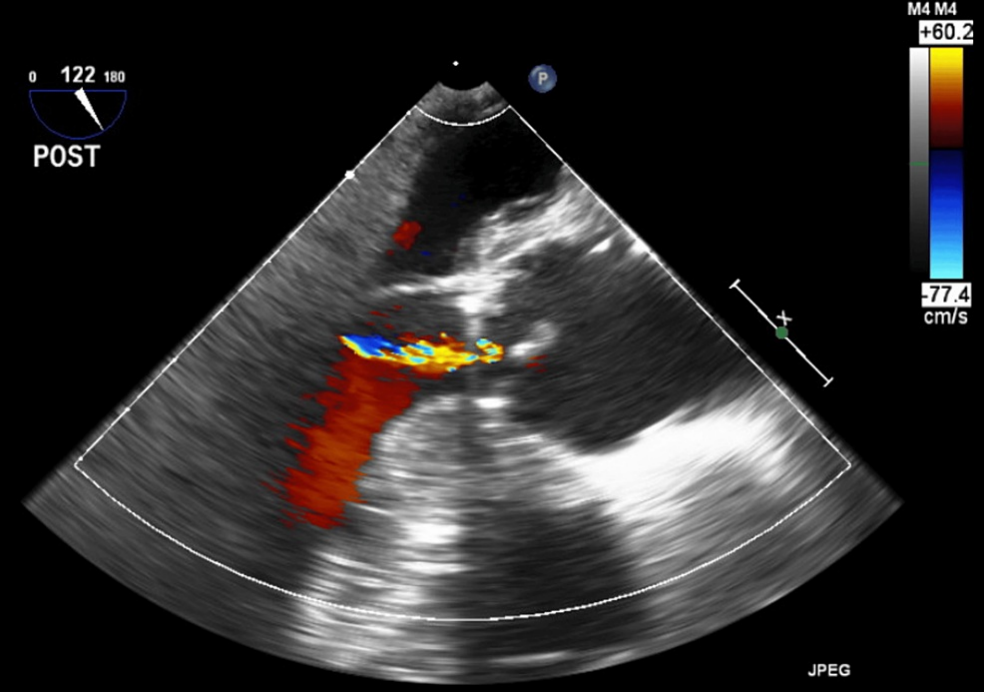
Post-bypass TEE imaging of the aortic valve revealing mild aortic insufficiency suggestive of successful repair

The intraoperative course was generally uneventful until chest tube placement and sternal closure. The patient remained hemodynamically stable throughout the course of induction, incision, initiation, and weaning of cardiopulmonary bypass. Post-bypass TEE revealed stable biventricular function with mild aortic insufficiency suggestive of successful aortic valve repair (Figure 1). 

After weaning from cardiopulmonary bypass, the patient had no quantitative or qualitative evidence suggestive of coagulopathy based on standard coagulation laboratory values and surgical assessment of hemostasis on the field. The surgeons then proceeded with chest tube placement and sternal closure. Following chest tube placement, the patient had a progressive increase in vasopressor support to achieve a mean arterial pressure (MAP) of 65 mmHg. The rise in vasopressor requirement was also associated with a slow but persistent downtrend in hemoglobin concentration, despite multiple red blood cell transfusions (Table 1). 

**Table 1:** Timeline of events following liberation from CPB CPB: cardiopulmonary bypass; INR: international normalized ratio; MAP: mean arterial pressure ^a^The surgical team began placing chest tubes in preparation for sternal closure approximately 30 minutes after liberation from CPB ^b^One hundred twenty minutes after liberation from CPB, the anesthesiology team informed the surgical team of a concern for ongoing bleeding in the setting of increased vasopressor requirement to maintain an MAP above 65 mmHg, downtrending hemoglobin concentration despite ongoing transfusions and TEE findings suggestive of intraabdominal bleeding ^c^One hundred eighty minutes after liberation from CPB, the patient underwent exploratory laparotomy once blood was identified in the abdomen via diagnostic laparoscopy ^d^The patient arrived to the intensive care unit approximately 270 minutes after liberation from CPB with stable vasopressor requirement and no further indication for blood transfusion

Minutes post bypass	0	30^a^	60	90	120^b^	150	180^c^	210	240	270^d^
Vasopressor infusion dose										
Norepinephrine (mcg/kg/min)	0.02	0.02	0.02	0.04	0.08	0.08	0.08	0.08	0.04	0.02
Vasopressin (units/min)	0	0	0	0	0	0.04	0.04	0	0	0
Epinephrine (mcg/kg/min)	0.02	0.02	0.02	0.02	0.02	0.02	0.02	0.02	0.02	0.02
Serum laboratory values										
Hemoglobin (g/dL)	8.5	7.8	8.5	7.8	6.8	7.9	8.2	8.8	8.5	7.7
Platelet count (k/mm3)		54					103			79
Fibrinogen (mg/dL)		254					277			254
Activated clotting time (seconds)	124	124	130	130			118			
INR	1.1						1.3			1.1
Number of transfusions administered										
Pack red blood cells (units)	0	1	0	2	2	2	0	0	0	0
Fresh frozen plasma (units)	0	2	2	2	2	2	1	0	0	0
Cryoprecipitate (pooled units)	0	0	0	0	0	1	0	0	0	0
Platelets (pooled units)	0	0	0	0	1	0	0	0	0	0

**Figure 2:**
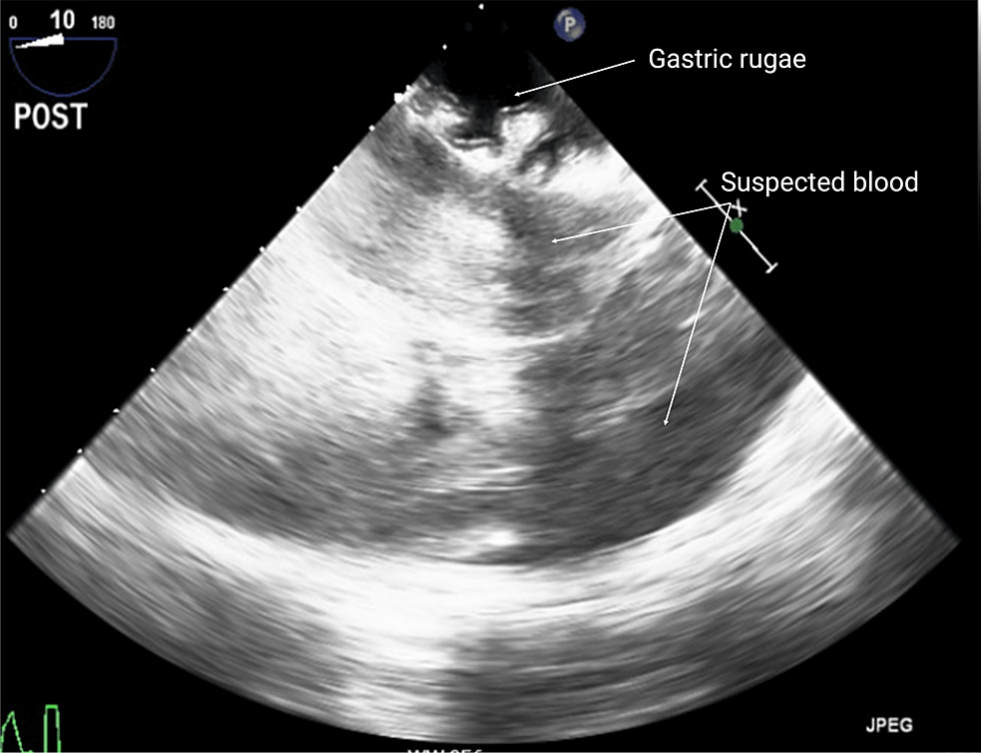
TEE view of the stomach revealing nonspecific hypoechoic areas anterior to the stomach concerning for blood accumulation TEE: transesophageal echocardiography

Upon further evaluation, chest tube output was minimal, and TEE evaluation did not identify any pericardial or pleural effusions suggestive of intraabdominal bleeding. Ongoing evaluation of the patient's cardiac function indicated no significant changes in cardiac function, although the ventricles did appear underfilled based on qualitative assessment. The TEE probe was then advanced into the stomach for further evaluation of the heart where there was incidental identification of nonspecific but distinct hypoechoic areas anterior to the patient's stomach, thought to be blood accumulation in the setting of the overall clinical picture (Figure 2). Although the TEE findings were subtle and generally nonspecific, the findings and concern for intraabdominal bleeding were discussed with the surgeons, who proceeded with skin closure but elected to consult general surgery for an evaluation of potential intraabdominal bleeding. 

Upon general surgery evaluation, the abdomen appeared soft, although examination was limited by the patient's body habitus. Focused Assessment with Sonography for Trauma (FAST) was performed but was inconclusive, again limited by the patient's body habitus. Diagnostic laparoscopy was performed which ultimately revealed blood in all four quadrants of the abdomen prompting exploratory laparotomy. Approximately one liter of blood was evacuated from the abdomen. Source control was achieved after bleeding was noted from a pre-peritoneal vein with associated peritoneal defect adjacent to a tunneled mediastinal chest tube with slow but persistent bleeding into the abdominal cavity. The patient's hemodynamics improved quickly without the need for additional blood transfusion and a decreased vasopressor requirement. 

The patient's abdomen was closed, and she was then taken to the intensive care unit for recovery in stable condition. The patient was extubated on postoperative day 1 and subsequently discharged to acute rehabilitation on postoperative day 13. She did not require additional transfusions and had an uncomplicated postoperative course.

## Discussion

Intraabdominal complications of open cardiac surgeries are rare, occurring in an estimated 0.3-3.0% of cases [3]. Furthermore, the use of TEE for identifying intraabdominal bleeding is even more rare, with only two prior case reports noted in the literature. One case identified intraabdominal hemorrhage during cardiac arrest in the setting of an intraabdominal surgery [4]; the other identified a retroperitoneal hemorrhage during cardiac surgery, particularly when placing femoral cardiopulmonary bypass cannula [5].

This case involved an insidious onset of hemorrhagic shock requiring a slow but consistent increase in vasopressor and transfusion requirement with inadequate response to transfusion. The lack of improvement in hemoglobin concentration following multiple transfusions was ultimately the clue that raised concern for ongoing bleeding. While the most obvious locations for bleeding were thought to be the pericardial or pleural spaces, given the nature of the surgery, the absence of any obvious pericardial or pleural effusions on TEE pushed the anesthesiologists to consider an intraabdominal source. The aorta was evaluated for potential dissection or perforation caused by bypass cannulation, which was not identified. Also considering the relatively slow, insidious onset of the suspected bleeding, it was unlikely to be a large-volume arterial bleed stemming from the aorta. While TEE provided a clue to the intraabdominal source of bleeding, there was no obvious explanation as to why this occurred until the abdomen was explored. Surgical exploration of the abdomen ultimately helped identify the source of bleeding from a pre-peritoneal vein which was injured by a tunneled mediastinal tube placed prior to chest closure.

Considering the level of hemostasis within the chest, prior to sternal closure and stable chest tube output, the surgical team was appropriately skeptical of any ongoing bleeding. As a result, clear communication was critical in achieving prompt evaluation and intervention during this case. A joint commission report from 2011 suggested that 56% of intraoperative and postoperative complications result from "communication failure," further emphasizing the importance of interdisciplinary communication in this case [6]. The patient's vital signs appeared stable on the monitors displayed throughout the operating room; therefore, the surgeons did not suspect any significant issues until the anesthesiology team raised concern for intraabdominal bleeding. While abdominal examination was limited, there was no obvious abdominal distention, further supporting the surgeon's initial impression. After thorough discussion of the overall clinical picture, including the TEE findings, the surgeons agreed to consult general surgery for intraoperative evaluation which confirmed the diagnosis of intraabdominal bleeding. Prompt identification of intraabdominal bleeding facilitated TEE, and open communication between perioperative team members spared this patient additional blood transfusions and an inevitable return to the operating room.

## Conclusions

This case required the anesthesiology team to use their entire skill set to help make a diagnosis that saved the patient from an inevitable return to the operating room and additional transfusions. Although the use of invasive monitors such as arterial lines, pulmonary artery catheters, and TEE probes has improved the anesthesiologist's ability to monitor surgical patients, effective communication of the data obtained from these monitors is key to helping guide intraoperative decision-making and patient care.
